# Follicular lymphoma-associated mutations in the V-ATPase chaperone Vma21 activate autophagy by dysfunctional V-ATPase assembly

**DOI:** 10.1080/27694127.2022.2077509

**Published:** 2022-05-29

**Authors:** Ying Yang, Zhihai Zhang, Daniel J. Klionsky

**Affiliations:** a Department of Molecular, Cellular, and Developmental Biology, University of Michigan, Ann Arbor, MI 48109, USA; b Life Sciences Institute, University of Michigan, Ann Arbor, MI 48109, USA

**Keywords:** Proton gradient, stress, vacuole, Vph1, yeast

## Abstract

A significant number of follicular lymphoma patients display recurrent mutations in subunits and regulators of the vacuolar-type H^+^-translocating ATPase (V-ATPase). Past studies focusing on the role of these mutations highlighted essential functions of macroautophagy/autophagy, amino-acid, and nutrient-sensing pathways in the pathogenesis of this disease. Here, we demonstrate novel results that help in understanding the role of the follicular lymphoma-associated hotspot mutation VMA21p.93X, which corresponds to Vma21[Δ66-77] in *S. cerevisiae* cells. We find that V-ATPase assembly is affected by the Vma21[Δ66-77] mutation, shown by decreased vacuolar levels of V_0_ subunits as well as a Vph1 stability assay. In addition, we report that vacuolar levels of histidine, lysine and arginine are significantly reduced in Vma21[∆66-77] mutant cells. These results deepen the current understanding on the mechanism of how autophagy is activated by these mutations in follicular lymphoma.

**Abbreviations:** DMSO, dimethylsulfoxide; ER, endoplasmic reticulum; FL, follicular lymphoma; V-ATPase, vacuolar-type H^+^-translocating ATPase; WT, wild-type.

## Introduction

The analysis of recurrent mutations associated with the vacuolar-type H^+^-translocating ATPase (V-ATPase) in follicular lymphoma (FL) have provided insight into the role of key homeostatic pathways in disease pathogenesis [1-4]. Recently, we reported that a somatic VMA21 hotspot mutation identified in tumor samples from FL patients, VMA21 p.93X, results in a partial defect in lysosomal acidification and proteolytic activity, along with elevated autophagy even though MTOR remains active [5]. This phenotype appears to represent a common adaptive mechanism for established tumors, allowing cell growth via active MTOR while providing essential nutrients through autophagy.

The V-ATPase is a proton pump composed of two sectors (V_1_ and V_0_). The peripheral V_1_ sector catalyzes ATP hydrolysis and the transmembrane V_0_ sector is responsible for proton translocation [6]. Vma21 is an essential assembly chaperone of the V-ATPase, primarily localized to the endoplasmic reticulum (ER). Vma21 is reported to have two critical functions: 1) Assisting in V_0_ assembly, and 2) escorting the assembled V_0_ subunits to the *cis*-Golgi where the V_1_ sector is assembled onto the V_0_ sector [7]. In addition, Vma21 contains a KKXX ER retrieval motif on its C terminus, which allows Vma21 to be transported back to the ER from the Golgi apparatus [8]. The VMA21 p.93X mutation results in the removal of the C-terminal ER retrieval signal, and therefore may lead to mislocalization of the V-ATPase.

In our prior study, to investigate the role of the VMA21p.93X mutation, we tested its effects in a yeast model. Sequence alignment showed that the VMA21p.93X mutation corresponds to yeast Vma21[Δ66-77]. Therefore, we generated a Vma21[Δ66-77] mutation in the genome. We showed that this FL-associated VMA21 mutation activates autophagic flux and causes compromised V-ATPase activity [5]. In human cells, we found that the levels of V_0_ and V_1_ subunits were not significantly changed. In addition, vacuolar amino acids levels displayed a disparity between the wild-type (WT) and mutant cells, being either higher or lower depending on the individual amino acid. However, the human aspect of our study is limited by existence of the endogenous WT VMA21. In contrast, the yeast Vma21[Δ66-77] mutation was generated in a haploid genome and is therefore not affected by WT Vma21. Accordingly, we extended our analysis of the yeast Vma21[Δ66-77] mutant.

In this study, we present our novel results showing decreased levels of the V_0_ subunit Vph1 in Vma21[∆66-77] cells compared to WT cells. We further confirmed that Vph1 is not correctly assembled in the mutant cells. Moreover, we determined that vacuolar amino acid concentrations were generally decreased. The phenotype we report in this study is slightly different from the previously published human study, deepening the understanding of FL-associated mutations, and therefore may provide further insight for innovative clinical trials in FL.

## Results

### The Vma21[∆66-77] mutation results in a decreased level of correctly assembled V-ATPase

**Figure 1.**
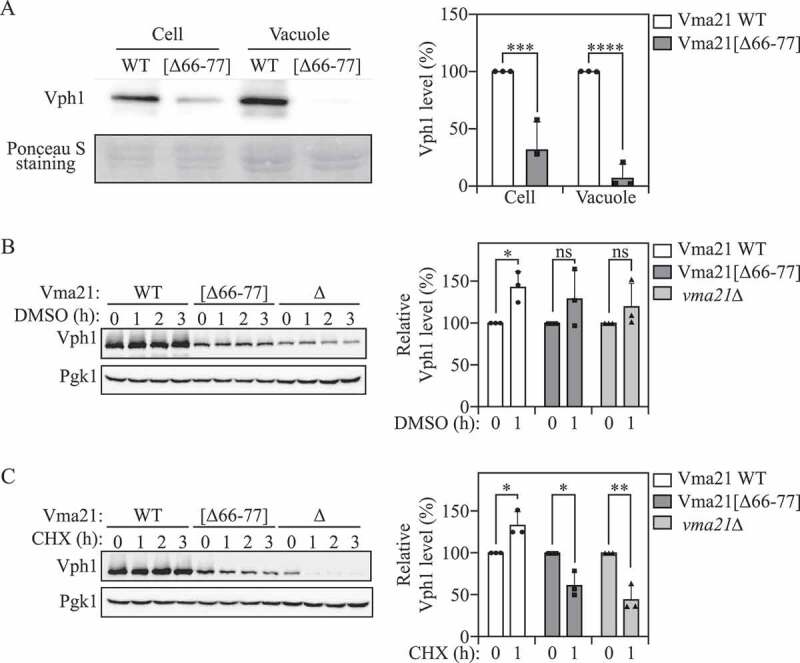
Vph1 stability is affected by Vma21[Δ66-77] mutation. (A) *S. cerevisiae* cells expressing either full-length Vma21 (WT Vma21) or a truncation mutant Vma21[∆66-77] were analyzed for assembly of V-ATPase subunits. The cellular and vacuolar Vph1 protein levels were monitored: The cells were grown in YPD to mid-log phase, and vacuoles were isolated. Cell and vacuole lysates were prepared and subjected to SDS-PAGE. The levels of Vph1 were analyzed by western blot. Ponceau S staining was used for total protein normalization. The quantitative analysis of cellular and vacuolar Vph1 protein levels is shown on the right. Either cellular or vacuolar Vph1 protein levels in WT cells were set to 100%, and the corresponding levels in Vma21[∆66-77] cells were normalized. (B) Vma21 WT, Vma21[∆66-77], and *vma21*∆ cells were grown in YPD to mid-log phase then shifted to fresh YPD with DMSO. Cell lysates were prepared, subjected to SDS-PAGE and analyzed by western blot. The ratio of Vph1 to Pgk1 was quantified. For all different Vma21 cells, the DMSO sample at the 0-h time point was set to 100% and other values were normalized. The quantification is shown on the right. (C) Vma21 WT, Vma21[∆66-77], and *vma21∆* cells were grown in YPD to mid-log phase then shifted to fresh YPD with cycloheximide (CHX). Cell lysates were prepared and analyzed as in (B). Mean±SD of *n*=3 independent experiments are shown as indicated. Unpaired, 2-tailed *t*-test; *: *p*<0.05, **: p<0.01, ***: *p*<0.005, ****: *p*<0.001, ns: not significant.

Previously, we found that vacuolar Vma2 levels are slightly lower in the Vma21[Δ66-77] mutant, and vacuolar Vma4 levels are not significantly different between the mutant and WT cells, similar to the result for human cells [5]. Vma2 and Vma4 are V_1_ subunits of the V-ATPase. To further investigate if the amount of V-ATPase V_0_ proteins in vacuoles was altered, we measured vacuolar protein levels of the V-ATPase V_0_ subunit Vph1, which is homologous to human ATP6V0A4. Vacuoles were purified from logarithmically grown yeast cells by dextran lysis and flotation, and subsequently western blot was used to detect vacuolar proteins. Both total cellular and vacuolar levels of Vph1 in Vma21[Δ66-77] cells were significantly lower compared to WT cells (Figure 1A). Prc1, a vacuolar carboxypeptidase Y that localizes to the vacuole lumen [9], was monitored to show the successful isolation of vacuoles at equivalent levels from both strains (Figure S1). Thus, there was an apparent discrepancy between the results for the V_1_ and V_0_ subunits.

Previous studies have shown that Vph1 can be rapidly degraded by nonvacuolar proteases when not fully assembled into the V-ATPase [8,10]. Therefore, examination of the turnover of Vph1 in different mutant strains can be used as an assay to test V-ATPase assembly function. Along these lines, we examined the stability of Vph1 using a cycloheximide chase. Vma21 WT, Vma21[∆66-77] mutant and *vma21*Δ (positive control) cells were treated with either DMSO (negative control) or cycloheximide to block protein synthesis, and protein extracts were examined for Vph1 levels by western blot. In the cells treated with DMSO, Vma21[∆66-77] mutant and *vma21*Δ cells showed lower total Vph1 protein levels compared to WT cells; there was a small increase in Vph1 at the 1-h time point in the WT strain and a similar increase in the mutants, the latter of which were not statistically significant. That is, in all three strains despite different starting levels Vph1 was essentially stable during the 3-h time course (Figure 1B). In contrast, following cycloheximide treatment we observed that Vph1 protein levels in the Vma21[∆66-77] mutant strain decreased significantly during the treatment compared to the WT strain while the Vph1 protein was rapidly degraded in the *vma21∆* strain (Figure 1C).

Taken together with our previous analysis of Vma2 and Vma4, these results indicate that the Vma21[∆66-77] mutation may not directly change the amount of V-ATPase subunits initially present on the vacuole, but instead may be more likely to result in a decrease in the total amount of correctly assembled functional V-ATPase as marked by the instability of Vph1. This phenotype would fit with the chaperone function of Vma21, and would explain the reduced V-ATPase activity and increased vacuolar pH detected in the mutant strain [5].

### The aberrant metabolome of Vma21[∆66-77] vacuoles is defined by amino acid profiling

**Figure 2.**
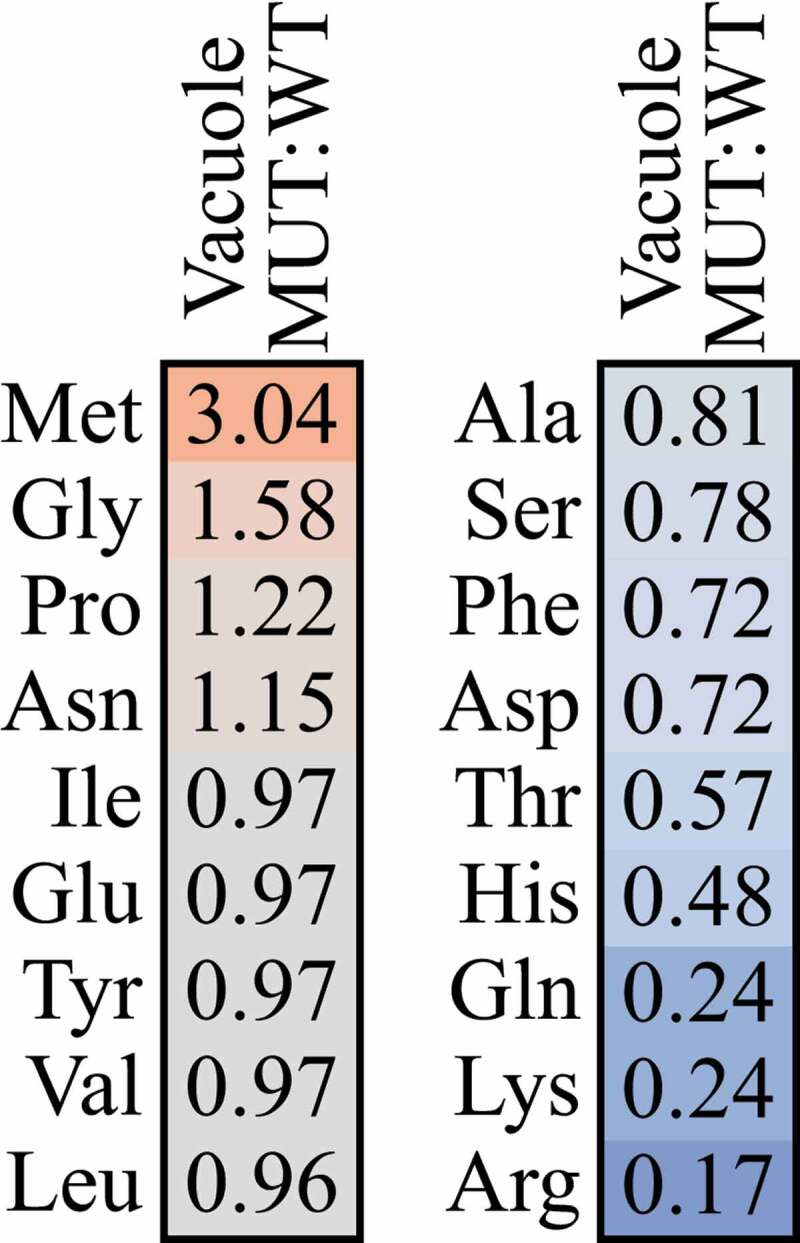
Analysis of amino acid levels in purified vacuoles. Fold-changes of vacuolar amino acid concentrations between Vma21[∆66-77] and WT cells. Targeted amino acids assays were performed on isolated vacuoles. Mean±SD of *n*=3 independent experiments are shown as indicated.

The yeast vacuole functions as a storage site for basic amino acids, and the ability to import these amino acids is dependent upon various proton antiporters in the vacuole [11]. To determine whether the Vma21[∆66-77] mutant affected the vacuolar import of amino acids, we measured amino acid concentrations from isolated vacuoles by a targeted assay and determined the fold-change of amino acid values between Vma21[∆66-77] and WT cells. Among the detected amino acids, histidine, glutamine, lysine and arginine were found to be significantly reduced in Vma21[∆66-77] mutant cells, suggesting that the mutants could not efficiently import amino acids for storage (Figure 2). In addition, the proteases in the vacuole display a pH-dependent activity [12]. Therefore, the reduced vacuolar pH seen in the Vma21[∆66-77] mutant may result in a concomitant reduction in vacuolar hydrolase activity; accordingly, the decrease in vacuolar amino acids may also be due to decreased vacuolar proteolysis.

## Discussion

The truncating nonsense mutation VMA21p.93X is present in 12% of DNA samples from FL patients. This mutation leads to impaired acidification and protein degradation in the lysosome and to a compensatory upregulation, and increased dependence on the autophagy pathway. Agents that disable activation of autophagy (e.g., ULK1 and potentially CDK inhibitors) are thus an attractive therapeutic strategy for treating these malignancies.

Deletion of *VMA21* in mammalian cells may be lethal, imposing certain limitations in the current study on VMA21p.93X. That is, it is technically difficult to introduce a mutation without endogenously expressing WT VMA21. Therefore, in the previous study, the VMA21p.93X mutation was created by transfecting the plasmid containing the mutated *VMA21* cDNA into WT lymphoma cells and the 293T cell line. The existence of the WT VMA21 protein may complicate our understanding of the role of this VMA21 truncation mutation. In contrast, *Vma21* is not essential in yeast, allowing us to analyze cells in which the mutant version of the protein is the only one being expressed. Consistent with findings using 293T cells, the Vma21[∆66-77] mutation leads to elevated autophagy flux, compromised V-ATPase activity, and increased vacuolar pH.

In this study, we find that the Vma21[∆66-77] mutation in yeast results in a decrease in the total amount of correctly assembled functional V-ATPase by affecting V_0_ sector assembly. This contrasts slightly from what was observed in 293T cells but may be explained by the presence of WT VMA21 in the latter. In addition, the Vma21[∆66-77] mutant may result in a concomitant reduction in vacuolar hydrolase activity, and a decrease in the proton gradient across the vacuolar membrane, both of which may account for the decrease in vacuolar amino acids.

## Materials and Methods

### Strains, media, and growth conditions

Yeast strain SEY6210 was used to generate the Vma21[∆66-77] mutation and *vma21*∆ in the genome using standard methods [13,14]. Specifically, Vma21[∆66-77] is a truncated form of the protein. The codon for amino acid 66 was mutated to a stop codon, which leads to premature termination of protein elongation. The Vma21[∆66-77] mutation was generated using the C-terminal deletion method as described previously [14]. Complete deletion of *VMA21* was performed using PCR-based methods [13]. Cells were cultured in rich medium (YPD; 1% [w:v] yeast extract, 2% [w:v] peptone], and 2% [w:v] glucose) as nutrient-rich conditions.

### Vacuole assays and amino acid profiling

Vacuole purification was performed as previously described [15]. Vacuole lysates were prepared and subjected to SDS-PAGE. Anti-Vph1 (Abcam, ab113683; 1:3,000 dilution) was used to detect the protein by western blot. A targeted amino acids assay was performed on isolated vacuoles at the Metabolomics Core at the University of Michigan using the EZfaast kit (Phenomenex, KG0-7165). Samples were extracted, semipurified, derivitized and measured by EI-GCMS using norvaline as an internal standard for normalization. The observed values for cysteine and tryptophan were below the noise level and therefore were not considered valid.

### Vph1 stability assay

Yeast cells were grown in YPD at 30°C until mid-log phase (OD_600_ = 0.8). For cycloheximide treatment, cells were centrifuged at 1500 g (4000 rpm) for 2 min and resuspended in fresh YPD with 100 µg/ml concentration of cycloheximide (Sigma, C4859), and then mixed briefly by vortex. For DMSO treatment, cells were centrifuged as above and resuspended in fresh YPD with DMSO (0.1% final concentration), and then mixed briefly by vortex. Samples were collected at the indicated times and used to generate protein extracts [10], followed by western blot [16] to detect Vph1 protein levels. The blot was imaged using a ChemiDoc Touch imaging system (Bio-Rad) and quantified using Bio-Rad Image Lab software.

### Statistical analyses

Statistical analyses were performed using GraphPad Prism 9. Statistical significance was determined from 3 independent experiments using Student’s *t*-test. *: *p*<0.05; **: *p*<0.01; ***: *p*<0.005. Number of independent experiments (n), statistical test utilized, dispersion of measurements and significance are described in the figure legends.

## Supplementary Material

Supplemental Material
