# Supplementary material for: Follicular lymphoma-associated mutations in the V-ATPase chaperone Vma21 activate autophagy by dysfunctional V-ATPase assembly
Source: Autophagy Rep. 2022 May 29;1(1):226–33. doi: 10.1080/27694127.2022.2077509 (PMC10309153; doi:10.1080/27694127.2022.2077509)
Supplement: Supplemental Material [file KAUO_A_2077509_SM1195.zip › Figure S1.pdf]

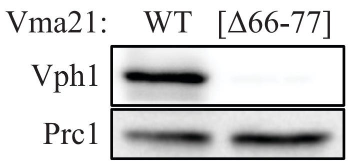

Figure S1. Protein levels for vacuolar marker proteins Prc1 and Vph1 in purified vacuoles from strains expressing wild-type Vma21 or Vma21[Δ66-77]. After vacuole preparation, vacuole lysates were prepared and subjected to SDS-PAGE. The levels of Prc1 and Vph1 were analyzed by western blot.
